# Hidden patterns among the fatally injured pedestrians in an Iranian population: application of categorical principal component analysis (CATPCA)

**DOI:** 10.1186/s12889-021-11212-x

**Published:** 2021-06-16

**Authors:** Milad Jamali-Dolatabad, Parvin Sarbakhsh, Homayoun Sadeghi-bazargani

**Affiliations:** 1 Road Traffic Injury Research Center, Tabriz University of Medical Sciences, Tabriz, Iran; 2 Department of Statistics and Epidemiology, Faculty of Health, Tabriz University of Medical Sciences, Tabriz, Iran

**Keywords:** Pedestrian safety, CATPCA, PCA, Traffic-related death, Iran

## Abstract

**Background:**

Identifying hidden patterns and relationships among the features of the Fatal Pedestrian Road Traffic Injuries (FPRTI) can be effective in reducing pedestrian fatalities. This study is thus aimed to detect the patterns among the fatally injured pedestrians due to FPRTI in East Azerbaijan province, Iran.

**Methods:**

This descriptive-analytic research was carried out based on the data of all 1782 FPRTI that occurred in East Azerbaijan, Iran from 2010 to 2019 collected by the forensic organization. Categorical Principal Component Analysis (CATPCA) was performed to recognize hidden patterns in the data by extracting principal components from the set of 13 features of FPRTI. The importance of each component was assessed by using the variance accounted for (VAF) index.

**Results:**

The optimum number of components to fit the CATPCA model was six which explained 71.09% of the total variation. The first and most important component with VAF = 22.04% contained the demographic and socioeconomic characteristics of the killed pedestrians. The second-ranked component with VAF = 12.96% was related to the injury type. The third component with VAF = 10.56% was the severity of the injury. The fourth component with VAF = 9.07% was somehow related to the knowledge and observance of the traffic rules. The fifth component with VAF = 8.63% was about the quality of medical relief and finally, the sixth component with VAF = 7.82% dealt with environmental conditions.

**Conclusion:**

CATPCA revealed hidden patterns among the fatally injured pedestrians in the form of six components. The revealed patterns showed that some interactions between correlated features led to a higher mortality rate.

## Background

Walking is one of the most affordable and healthiest ways to travel in developing countries [1]. However, pedestrians are among the most vulnerable road users [2], which about 22% of 1.35 million fatalities due to traffic injuries in the world are related to them [3].

According to a study assessing the pedestrian mortality rate in the world, this rate has decreased by 28%, mainly in the high and middle-income countries, but no significant reduction has been observed in low-income countries [4].

Pedestrian injuries are declining in developing countries due to their effective interventions, while this rate is still high in developing countries reflecting a deep gap in the knowledge required for traffic accident safety, especially for pedestrians [5, 6].

According to the World Health Organization (WHO), Iran is one of the developing countries with a high rate of traffic-related death. The estimated road traffic mortality rate in Iran is 20.5 per 100,000 people in 2016; 23% of this rate is related to pedestrians [3]. The rate of pedestrian fatality due to traffic injuries is high in populated cities [7]. East Azerbaijan is located in the northwest of Iran. According to the Iranian population census in 2016, the population of this province is 3.91 million, accounting for 4.89% of the country’s total population; thus this province is one of the most populated ones with a high rate of Fatal Pedestrian Road Traffic Injuries (FPRTI).

The pattern of fatal traffic injuries can be different from non-fatal ones. Also, it can be different in various places. So, identifying patterns of the injuries data and information on the importance and priority of the contributing factors are of utmost significance. Recognition of such patterns enables managers and policymakers to estimate the effectiveness of interventions designed to reduce injuries or the severity of injuries.

On the other hand, many variables (in machine learning terminology; features) contribute to the occurrence and severity of traffic injuries. In data with high dimensions, it could be hard to manually detect and describe patterns. So, accurate pattern extraction requires a better approach to deal with high-dimensional data for simpler interpretations. Dimension reduction techniques can reduce a large set of variables to a smaller one which still contains most of the information. This approach offers the possibility of quick extraction of patterns and insights. One of the most commonly used methods to reduce the dimension of data and reveal hidden patterns is the principal component analysis (PCA). The nonlinear categorical principal component analysis method (CATPCA) is an extended version of this method for categorical data (i.e., ordinal and nominal data) [8].

The objective of this study is to identify the hidden pattern among the fatally injured pedestrians due to the road traffic injures in East Azerbaijan, Iran from 2010 to 2019 based on the forensic organization data using the CATPCA method for taking into account the unsupervised nature of the data (unlabeled data) and categorical nature of the variables.

This research is part of a major project of “National Document for Health and Traffic Safety in the Islamic Republic of Iran” from 2021 to 2030 to develop the vision, goals, and strategies for Health and Traffic Safety [9].

## Methods

### Data

The present study is a cross-sectional (descriptive-analytic) study based on data collected by the forensic organization of East Azerbaijan province. According to the WHO definition, a fatal traffic injures was defined as the injures in which the person involved in the traffic accident was killed immediately or within 30 days as a result of the accident [3]. Inclusion criteria: A total of 7785 deaths due to the fatal road traffic injures has been recorded by the forensic organization of East Azerbaijan province during the years 2010 (March 21) to 2019 (March 21). Exclusion criteria: From them, 139 injuries had occurred in other provinces, and 238 deaths had occurred after the 30th day so they were omitted from the data. Therefore, according to the WHO definition, 7408 traffic injures fatalities have occurred in East Azerbaijan province of Iran which 1782 of them (24.05%) were the FPRTI.

So, the final number of FPRTI that were included in this study was 1782. Collected data of the forensic organization includes information such as demographic and socioeconomic characteristics of the pedestrians with fatal injuries due to the road traffic (age, gender, job, marriage, and education), kind of vehicle involved in the crash, type of the transferring pedestrians with fatal injuries to hospital, injured organs, leading cause of death, location of the death, location of the accident (urban- Suburban roadways), lightness condition and season of the accident. Details of data collection have been published elsewhere [10].

### Statistical methods

To describe the data numbers and percentages were used. Association between variables was assessed via the Chi-Square test. To find the structure and principal components of the data, due to the categorical nature of our variables (ordinal or nominal), the unsupervised CATPCA method was used for data analysis. Due to the low missingness rate, cases with missing values on each variable were excluded from the CATPCA analysis.

In the following, a brief introduction to the CATPCA method is mentioned.

### The PCA and CATPCA methods

In scientific research, summarizing and extracting information from raw data is very important. Considering that in recent decades the data collected in all areas, especially in the field of medical sciences, have high dimensions, statistical methods in extracting information from these data become problematic.

One of the statistical methods used in this situation is the dimension reduction technique. So that the information in the total data is summarized in some components that are derived from the combination of the main variables so that extracted components still contain most of the information of the original data. In practice, instead of using all the variables in the analysis, extracted components are used.

One of the most commonly used methods for dimension reduction is the principal component analysis (PCA) method that is an unsupervised reduction technique. In unsupervised methods, the users do not need to supervise the model; instead, it allows the model to work on its own to discover patterns and information that was previously undetected. The purpose of PCA, as an unsupervised method, is to reveal hidden structures and bring out strong patterns in a dataset by converting a set of observations of possibly correlated variables into a smaller number of uncorrelated variables called principal components and rank these components based on the score of each component.

The PCA method begins this task by estimating the Sums of Squares and Cross Products matrix (SSCP matrix = *X*^*T*^*X*) of the predictor variables.

In linear algebra, an eigenvector of matrix A is a nonzero vector v so that Av = λv, for some scalar λ. An eigenvalue of A is a scalar λ such that the equation Av = λv has a nontrivial solution. If Av = λv for v ≠ 0, λ is called the eigenvalue for v, and that v is an eigenvector for λ.

The base of PCA is established on the notion of Eigenvectors and Eigenvalues of SSCP. The SSCP matrix is the covariance matrix without subtracting the mean. The diagonal values are sums of squares and the other values are sums of cross products. The eigenvalues and eigenvectors of the SSCP matrix S are constructed. The eigenvectors of the SSCP named the principal components of X.

In the PCA method, instead of using all the Eigenvalues, such as the regular regression method, we try to extract and apply a smaller number of Eigenvalues [11].

The non-linear CATPCA method is the nonlinear equivalent of the PCA method to reduce dimensions in categorical data. The most important advantage of non-linear CATPCA over the linear PCA method is that it combines nominal and ordinal variables and can discover nonlinear relationships between variables. Unlike the PCA method, CATPCA does not have high sensitivity to classical statistical assumptions such as normality (multivariate normality) and linear relationships between variables [8, 12].

CATPCA converts every category of the variables to a numeric value, using optimal quantification (also known as optimal scaling). In CATPCA similar to PCA, the overall summary diagnostic value is the percentage of variance accounted for (VAF) by the principal components which equals the sum of the eigenvalues (sum of squared of loading value of constituent variables) of the components divided by the total number of variables [12].

The importance of each component was assessed by using the variance accounted for (VAF). Scree plot and Kaiser’s criterion which recommends retaining all factors with an eigenvalue above 1 [12] were used to determine the optimum number of components. Varimax with Kaiser Normalization rotation method was used to rotate components. The R software version 3.5.1 and Gifi packages were used to fit the CATPCA model.

## Results

### Data description

In this study, data of 1782 FPRTI were investigated. Thirteen features related to these injuries were included in the data analysis.

The descriptive pattern among pedestrians with fatal injuries was as follows: Of the 1782 pedestrians with fatal injuries, the majority of victims were male (78.23%). with increasing age, the number of victims increased, so that, most of the victims (77.84%) were over 30 years old and (35.13%) were over 65 years old.

Most pedestrians with fatal injuries who lost their lives in traffic injuries (75.08%) were married. Moreover, the majority of them were illiterate or had primary education (69.64%) and self-employed (52.52%). Although there was no major difference in the number of pedestrians with fatal injuries in different seasons, the highest number of fatal injuries occurred in the summer (29.24%) while winter had the lowest rate (20.15%). In 72.22% of pedestrians with fatal injuries, a light vehicle was involved in the accident. Most fatal injuries (62.40%) occurred in daylight, and most of the victims (79.18%) were taken to the hospital by ambulance. Also, in most of these fatal injuries, injured organs were the head and neck (52.3%) and the most common leading cause of death was head trauma (58.25%). Furthermore, 40.7% of traffic-related pedestrian fatalities occurred at the scene of the accident, and 50.95% of the deaths happened in the hospital or after discharge from the hospital; the remaining cases died during transfer. The majority of fatal injuries (59.82%) occurred on suburban roads.

### The results of the CATPCA method

**Fig. 1:**
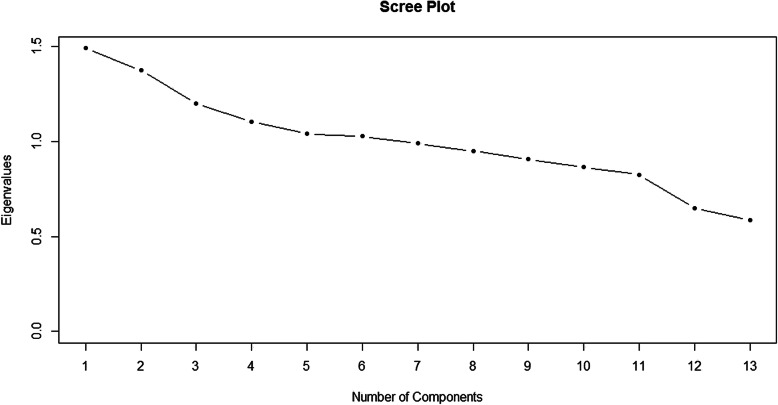
The scree plot

For pattern extraction by CATPCA and finding hidden relationships among variables, ordinal and nominal variables were defined in the model. Given that the maximum number of possible components is the number of the main variables, the initial model was fitted using 13 components to determine the optimal number of components. Figure 1 shows the scree plot of the data, the point where the slope of the curve is leveling off (the elbow) was six, indicating the number of factors that should be generated by the analysis. Also, according to Kaiser’s criterion to find the number of the major components (with Eigenvalue higher than 1), the most appropriate number of components in this study was six.

Therefore, the CATPCA model with six components was fitted to the data. All underlying variables remained in CATPCA analysis with considerable loading values (> 0.4). The results are presented in Table 1.

**Table 1:** Loading value of the features loaded in the six major components

Features	Comp1 (demographic and socioeconomic characteristic)	Comp2 (injury)	Comp3 (severity of the accident)	Comp4 (knowledge and observance of the traffic rules)	Comp5 (medical relief)	Comp6 (environmental conditions)
Age group	**0.944**	0.060	0.054	−0.027	− 0.089	0.073
Marital status	**0.910**	0.056	0.025	−0.139	−0.065	0.098
Pedestrian occupation	**0.879**	0.012	0.079	0.074	−0.027	0.031
Injured organs	0.046	**0.908**	0.010	0.008	−0.062	− 0.008
The leading cause of death	0.054	**0.906**	−0.062	0.012	0.058	−0.002
Type of vehicle involved	0.106	−0.127	**0.802**	0.003	0.066	−0.030
location of the death	0.001	0.109	**0.675**	0.021	−0.389	0.015
Education	−0.098	0.016	−0.081	**- 0.770**	−0.194	0.185
Gender	−0.161	0.036	−0.073	**0.675**	−0.217	0.144
Location of accident occurrence	−0.081	0.009	−0.194	−0.063	**0.800**	−0.035
Transmission of the injured	−0.338	0.008	0.170	0.301	**0.418**	0.138
Season of the accident occurrence	0.105	0.003	−0.188	0.168	−0.199	**0.756**
Darkness	0.078	−0.018	0.204	−0.234	0.267	**0.710**

Rotation Method: Varimax with Kaiser Normalization

According to Table 1, the information in the data can be summarized in six components. These six components together explain 71.09% of the total variation of the data which includes an acceptable amount of total variance.

Based on Table 1, “age”, “marital status” and “job” of the fatally injured pedestrians were the first and the most important principle component with the largest Eigenvalue (2.86) and consequently the largest amount of the explained variance (22.04%) (Table 2). We can name this component as “demographic and socioeconomic factor”.

**Table 2:** Eigenvalues values and variance explained by each component

	Comp1	Comp2	Comp3	Comp4	Comp5	Comp6
Eigenvalues	2.86	1.68	1.37	1.18	1.12	1.06
variance accounted for (VAF)	22.04	12.96	10.56	9.07	8.63	7.82
Cumulative VAF	22.04	35.00	45.56	54.63	63.26	71.09

About the hidden pattern and correlation of these variables with each other that appeared in the first component and formed demographic and socioeconomic factor in FPRTI data, the results showed that significant pairwise associations between all these three variables (*p*-value< 0.001). The pattern was that in the aged > = 30 years fatally injured pedestrians, in both single and married marital status, the majority of victims were self-employed. This rate in single pedestrians with fatal injuries was 62.5% and in married killed pedestrians was 60.9%.

According to Tables 1 and 2, the second most important component with 12.96% of explained variance was “the injured organ” and “leading cause of death” which was named “injury”.

About the hidden relationship and correlation between these two variables forming injury type component, there was a significant association between them (*p*-value< 0.001) and the results show that the rate of head trauma as a leading cause of death is higher in the killed pedestrians with head injury (87.3%) in compared to other injured organs.

The constituent variables of the third component were “the type of vehicle” and “the place of death”. With VAF 10.56%, this component was in the third rank of importance. This component was labeled as injury severity. The hidden relationship between these two variables was statistically significant (*p*-value< 0.001). The rate of death at the injury sense was higher in injuries with heavy vehicles (56.6%) as compared with light vehicles (38.1%).

The fourth component consisted of “education and gender of the fatally injured pedestrians” which also explained 9.07% of the total variance. The hidden relationship between these two variables was statistically significant (*p*-value< 0.001), reflecting their correlation. About the hidden relationship and correlation of these two variables, the result showed that 38% of female victims were literate while this rate was 59.9% in male victims. It seems that interaction between gender and education is related to the knowledge and the observance of the traffic rule. So we named this component “knowledge and observance of the traffic rules”.

The fifth component was related to “the location of the injury” and “mode of transferring fatally injured pedestrians to the hospital” which explained 8.63% of the total variance of the data and dealt with the quality of medical relief.

The relationship between these two variables was significant (*p*-value< 0.001). The result showed the higher rate of transferring pedestrians with fatal injuries to hospital by ambulance in the suburban road (89.8%) as compared to urban roads (82.4%).

Finally, the last component involved “season” and “light condition of the injury”. With VAF =7.82%, this component was about environmental conditions. Concerning the hidden pattern, the relationship between these two variables was statistically significant (p-value< 0.001). Most of the daylight FPRTI occurred in the summer (31.1%) and spring (28.1%). Also, most of the FPRTI occurring in twilight hours were in autumn (41.9%) and winter (28.1%). About night injuries, there was no significant difference in the rate of fatal injuries between seasons.

## Discussion

This research was conducted to identify the hidden pattern among the fatally injured pedestrians due to the road traffic injures in East Azerbaijan during 2010–2019. Concerning the ordinal and nominal nature of the variables, CATPCA was the most appropriate method to extract the pattern of this data and hidden relationships among variables. Identifying patterns in the data of the pedestrians with fatal injuries crashes and the importance and priority of the factors affecting fatal traffic injuries could effectively reduce injuries or the severity of injuries. Furthermore, the use of powerful statistical methods such as CATPCA in traffic data analysis can help the researchers to improve the statistical capacity of studies and the accuracy and precision of their findings to extract the hidden aspects of the data.

According to the results of the descriptive pattern, most of the FPRTI victims were male, elderly, self-employed, illiterate, and married. Also, pedestrians with fatal injuries in the summer were slightly more common. Furthermore, most of the fatal injuries occurred during the day, on suburban roads, and with light vehicles. In most FPRTI, the injured victims were taken to the hospital by ambulance. Also, in most of these fatal injuries, injured organs were the head and neck and the leading cause of death was head trauma. These findings were almost similar to the other studies [13].

Concerning the hidden patterns, six underlying variables in CATPCA analysis with considerable loading values explained 71% of the total variation in the data. This considerable amount of explained variance demonstrates the ability of this model to summarize information of these high-dimension data.

Regarding the unsupervised nature of the data and lack of outcome variables, the fatal injuries features distribution was compared to their distribution in the target pedestrian population or pedestrian injuries population to assess and discuss influential factors related to the FPRTI. The difference shows that these features affect the incidence of an injury or its severity or both.

The first and most important component was the demographic and socioeconomic characteristics of the pedestrians with fatal injuries (age, job, marriage). It can be concluded that the features in the first component are more focused on pre-crash characteristics which influence the incidence and severity of the crash.

Regarding the age itself, according to the studies, age has an undeniable effect on occurring an accident or its severity or both of them due to the lack of ability of the children and elderly age groups or the risk-taking nature of young age groups. It has been observed in a number of studies that age has a direct relation with the death of pedestrians, and older people are more likely to die [14–17]. In this study, the results indicated that the number of fatalities increased by age e.g. the 35.13% of victims were over 65 years of age.

Occupation and marital status were effective in the traffic volume on the road. The self-employed group composed the majority of the killed pedestrians. The possible explanation for this might be due to the fact that the self-employed pedestrians are one of the major users of the road and have more traffic volume in comparison to the occupational groups. Moreover, this occupation group usually has a lower level of education compared with other occupational groups, which might be a risk factor for the higher vulnerability of this group. Consistent with other studies, pedestrians with a self-employed occupation were more likely to have fatal traffic injuries because of more traffic on the road [18, 19]. Regarding the pattern, although due to the nature of the marital status, age, and occupation, the existence of an association between these variables was expected, some parts of these associations were different from the usual population.

In the aged > = 30 years pedestrians with fatal injuries, both single and married, the majority of victims were self-employed. Although most Iranian people are self-employed, the observed percentage is higher than the self-employed people rate in the Azerbaijan population. It shows that being self-employed can lead to a higher risk of FPRTI due to the higher volume of road traffic.

The second-ranked component was the injured organ (injured organ and the leading cause of death). This component is a post-crash factor related to the severity of the injury. There is no doubt that the injured organs and the leading cause of death are indicators of the severity of the injury with an important role in the outcomes [13, 19–21]. About their hidden relationship, the rate of head trauma as the leading cause of death is higher in the killed pedestrians with head injury.

The third component dealt with the severity of the injury (the type of vehicle, place of death). The type of vehicle is a factor influencing both the occurrence and the severity of the injury. In terms of occurrence, it can be due to the higher number of light vehicles and therefore the higher volume of road traffic (which leads to a higher number of traffic injuries). Concerning severity, upon an accident, the chance of death is higher for a heavy vehicle because of the shape, mass, and design of the car that increase released kinetic energy of the accident [14, 15, 22–24]. Also, the studies have shown that crashes involving motorcycles were less likely to die [25, 26]. Concerning vehicle type, the contribution of heavy vehicles in fatal injuries was more than their proportion in the non-fatal pedestrian injuries [27]. Consequently, the proportion of light vehicle (76.1%) and motorcycle or bicycle (5.9%) will be less than their proportions in non-fatal injuries [27].

The location of death can also be an indication of the severity of the incident which can occur at the injury sense or in the hospital or after discharge, depending on the severity of the incident. In this research, about half of pedestrians with fatal injures died in the hospital or after discharging and about 40% of them lost their lives at the injury sense; others died on their way to the hospital.

Between 2006 and 2012, 159,227 people died in traffic injuries in Iran. A total of 97,336 (61.13%) of them died inside hospitals or other health centers and 61,891 (38.87%) of the cases died at the injury scene or during the transfer [28] which is consistent with our result.

About the hidden relationship and correlation between the type of vehicle and place of death, the result shows that the rate of death at the accident sense is higher in injuries with heavy vehicles (56.6%) as compared with light vehicles (38.1%) which could be assigned to the severity of the injury.

The fourth component was related to the knowledge and observance of the traffic rules. According to the results, the majority of the killed pedestrians were men (78.23%). Because the gender distribution of the under investigation data is not similar to its distribution in East Azerbaijan population (50% male, according to the last census) and also, is not similar to its distribution in non-fatal pedestrian injuries in East Azerbaijan and is similar to its proportion in the fatal injuries [27], consistent with other studies [15, 23, 29, 30] it can be concluded that the gender is an effective factor on both occurrence and severity of the injuries . Concerning the incidence, the rate of traffic injuries was higher in males which can be due to more road traffic volume in males than females (maybe because of the type of their job) and lower observance of the traffic rules and risk-taking behaviors in males.

After the incidence of an injury, the severity of that injury can be higher for males than females probably due to their crossing in an unauthorized hazardous location such as highways. The high-speed collisions on highways lead to higher severity injuries.

On the other hand, education plays an important role in the occurrence and severity of injuries. According to the results, most of the victims were illiterate (44.9%). This rate is completely different from the rate of illiteracy in East Azerbaijan (15%) according to the last census. Thus, it can be an effective factor in the incidence and the severity of an injury. This finding is consistent with other studies [19]. A combination of these two risk factors (gender and education) led to the groups of pedestrians with fatal injuries that have the lowest and highest mortality rate. The majority of the victims were illiterate male pedestrians with fatal injuries (31.4%) while the minority were females with an academic education (0.5%). About the hidden relationship and correlation of these two variables, the result showed that 40.1% of male victims versus 62% of female victims were illiterate while the proportion of illiteracy in the East Azerbaijan population is 10% of males versus 20% of females.

So we can be concluded that education is more effective in the females’ awareness and observance about traffic rules as compared to males. The highly educated women usually tend to act more lawfully and safely.

The fifth component was about the quality of medical relief (place of injury, transferring by ambulance). According to the results, most of the fatal injuries occurred on suburban roads.

The Suburban incident is usually more fatal due to lack of quick relief to injured people as compared with an urban injury or due to the high-speed of vehicles or higher traffic of the heavy vehicles on the roads outside of the city [25, 29, 31, 32].

Furthermore, high severity of injury due to the high-speed of vehicles on the out-of-town roads or higher traffic of the heavy vehicles on the roads outside of the city rather than inside could be the cause of high mortality rates in out-of-town events. This issue has been assessed in many studies and it has been shown that the chance of death in events occurring in the urban areas and high-density sites is lower than out-of-town roads due to the severity of the injuries [29, 31, 33, 34].

Regarding the transferring of the injured person, it is obvious that transferring an injured person to a hospital by ambulance can reduce the fatalities. Between 2006 and 2012, 159,227 people died in traffic injuries in Iran. A total of 97,336 (61.13%) of them died inside hospitals or other health centers and 61,891 (38.87%) died at the accident scene or during the transfer [28]. The results showed that the majority of the killed pedestrians have been transferred to hospital by ambulance (79.18%) indicating the appropriate medical relief facility. However, about 21% of victims were transported by a vehicle other than an ambulance, reducing this number to zero can be effective in reducing pedestrian road traffic deaths.

Concerning the hidden relationship and correlation of place of injury and transfer by ambulance (i.e. quality of medical relief component), the result shows that the rate of transferring pedestrians with fatal injuries to hospital by ambulance was higher in the suburban road (89.8%) as compared with urban roads (82.4%). This means that the victims are more transferred to hospitals by other cars in the cities which can be assigned to the proximity to hospitals and more abundance of other cars in the injury sense. This type of transfer, however, increased the mortality rate.

The sixth component was about environmental conditions (time of day and season of the injury). The season of the injury can also affect both the occurrence and severity of the injury [35–37].

In most studies, it has been observed that injuries occurring at night or lower lighting conditions have a direct relationship with the severity of the incident, as the severity of the injury rises in low light conditions [25, 26, 31, 34, 38]. In this study, most of the fatal injuries occurred in summer and daylight which might be due to the higher traffic load of the pedestrians on the roads during the day and summer. Also, due to favorable weather and light conditions, drivers usually drive carelessly at higher speeds, making it less possible to control the car and prevent injuries. In terms of the hidden patterns, the results suggested a strong association between the outcome of traffic injuries and environmental conditions. Most of the daylight pedestrians with fatal injuries occurred in the summer and spring seasons. Also, most of the pedestrian fatal injuries in twilight hours were in autumn. For night injuries, there was no significant difference between seasons. It can be due to the more number of fatal pedestrians injuries during the day in the summer as well as the higher speed of cars in this season due to the favorable weather condition.

The changes in day duration, light, and weather conditions in the twilight hours of the autumn season can enhance the risk of injuries.

Investigation of hidden patterns and relationships among variables showed that in addition to the main effects of the risk factors on the occurrence and severity of the injury, there are some combinations of features such as age, employment condition, sex, or education level which can affect the risk of occurring or severity of an injury. In other words, older, male, self-employed, and illiterate pedestrians are more prone to fatal injuries. Identification of such combinations can help in focusing on details of such certain groups of pedestrians not all people to design more effective intervention programs. Also, injuries with heavy vehicles which resulted in head injury were more fatal. Furthermore, not transferring injured pedestrians by ambulance in urban areas, daylights of summer, and twilights of autumn increased the risk of fatal injuries. So, improving the lighting condition of the road and improving the relief facilities and car design can decline the victims of fatal traffic injuries.

### Limitation

The limitations of this study were the low number of variables collected by forensic medical information registration system and the lack of links among other relevant databases, such as the police or the hospital databases to collect or complete the related variables more precisely.

This issue has led to the loss of useful information such as cause of the crash (speeding / under the influence / unmarked crossing, hospital information etc) for pattern of injuries recognition.

## Conclusions

CATPCA revealed some hidden patterns among the fatally injured pedestrians due to the road traffic injures in the format of six components. It shows that some combinations of features can increase the risk of having a fatal injury.

Thus, identifying and controlling such hidden patterns according to their importance could be effective to reduce the occurrence and severity of pedestrian traffic injuries in East Azerbaijan province of Iran. Following this paper, it is suggested that similar studies be performed on FPRTI data of different places to compare their patterns.

## Data Availability

The datasets analyzed during the current study are not publicly available due used data was registered in the forensic organization and they do not belong to the researchers of this study but are available from the corresponding author on reasonable request.

## Abbreviations

FPRTI: Fatal Pedestrian Road Traffic Injuries
CATPCA: Categorical Principal Component Analysis
PCA: Principal Component Analysis
VAF: variance accounted for
